# Racial and Ethnic Variation in the Association of Social Integration with Mortality: Ten-year Prospective Population-based US Study

**DOI:** 10.1038/srep43874

**Published:** 2017-03-06

**Authors:** Steven D. Barger, Bert N. Uchino

**Affiliations:** 1 Department of Psychological Sciences, Northern Arizona University, 1100 South Beaver Street, Building #60, Room #338, Flagstaff, AZ 86011, USA; 2 Department of Psychology and Health Psychology Program University of Utah Salt Lake City, UT 84112, USA.

## Abstract

Substantial data link social relationships with mortality but few studies have examined whether these associations are consistent across racial and ethnic groups. The purpose of the present study was to evaluate the presence and form of the social relationship/mortality association in a representative sample of US Black (*n* = 4,201), non-Hispanic White (*n* = 20,217) and Hispanic (*n* = 5,097) groups. In models adjusted for age, sex, chronic disease, socioeconomic status and smoking social integration was inversely related to ten-year survival in all groups. However, among Whites the association was linear and graded whereas among Blacks the association was linear but was statistically significant only for the highest level of social integration (hazard ratio [HR] = 0.66, 95% confidence interval = 0.47–0.94). A threshold pattern was observed among Hispanics, in that lower mortality risk was found for all social integration categories above the lowest level (HRs from 0.58 to 0.52, P’s < 0.01) and each of the higher social integration categories were in turn equivalent. Received social support was unrelated to mortality across all groups. Higher social integration is associated with a survival advantage for Blacks and Whites. For Hispanics, moderate and high levels of social integration were equally protective.

The quality and quantity of one’s social relationships is strongly associated with all-cause mortality^1^,^2^,^3^. In a meta-analysis of 148 studies comprised of over 308,000 participants, those with more frequent social ties had a 50% lower mortality risk relative to those with fewer (or poorer quality) social relationships^2^. Indeed, links between relationships and all-cause mortality appear comparable to standard biomedical risk factors including smoking and physical activity^2^.

Although the literature provides consistent evidence for this association, several questions remain. First, it is unclear whether the generally linear social relationship/mortality association is also present within major US racial and ethnic subgroups, i.e., Blacks and Hispanics. Evaluating the presence and form of this association in minority groups is important to verify theoretical assertions that social relationships are associated with better physical health^1^,^4^ and to contextualize broad claims regarding the salutary health consequences of social relationships^2^. These widespread claims would be challenged to the extent that the social relationship/mortality association varies by race/ethnicity.

There is some evidence suggesting that social relationships are patterned differently by race and ethnicity. For example, one study found that social networks were inversely associated with all-cause mortality among Whites but not Blacks^5^. However, differences in the statistical association within these groups (which is relative to a null hypothesis) do not identify risk equivalence between groups and a direct comparison of hazard ratios^6^ for Black and White participants revealed them to be statistically equivalent. In a study of elderly Hispanics researchers found a threshold association whereby decreased all-cause mortality risk was observed similarly among all participants not in the lowest social support category^7^. Thus, evidence to date suggests that social relationships are similarly protective among Blacks and Whites but that social connections beyond those of the most socially isolated may not confer additional survival advantage among Hispanics. More research is needed to understand these patterns, particularly studies utilizing contemporaneous cohorts of Black, White and Hispanic participants. The present study addresses this gap by comparing the social relationship/mortality association across these groups in a diverse, nationally representative US cohort followed for ten years.

A second important question is whether the putative social relationship/mortality association is independent of socioeconomic status (SES). SES indicators such as education, wealth, work force participation, etc. are inversely associated with mortality^8^,^9^,^10^,^11^,^12^ and Blacks and Hispanics in the US have fewer of these resources relative to non-Hispanic Whites^13^,^14^,^15^. Thus, particularly for racial and ethnic minorities, SES-attributable health disparities are a potent rival explanation for any social patterning of mortality risk. We therefore included these key SES indicators to more precisely partition the unique contribution of social relationships to mortality risk.

The present study evaluated the presence and form of the social relationship/mortality association in a nationally representative sample of US Blacks (*n* = 4,201), non-Hispanic Whites (*n* = 20,217) and Hispanics (*n* = 5,097) who participated in the 2001 US National Health Interview Survey (NHIS). A prior report using NHIS data showed a linear inverse association of social integration with five-year mortality risk^16^. However, there were insufficient mortality events at five years to model the association within racial and ethnic groups. A subsequent ten-year vital status ascertainment^17^ provides a unique opportunity to model survival separately for each of these three social groups in a prospective, nationally representative sample. The purpose of this study was to examine whether the presence and form (linear, threshold) of the social relationship/mortality association is consistent across Black, White and Hispanic groups. To address whether this association was independent of SES, we statistically controlled for four SES indicators that are established predictors of mortality at the population level^8^,^9^,^10^,^11^,^12^ and that provide coverage of a broad range of SES domains^18^.

## Methods

### Data source

We analyzed data from the 2001 NHIS, a nationally representative survey of noninstitutionalized US residents (see ref. 19 for a detailed description of the NHIS design). Households are selected via probability sampling and an adult household member is selected and interviewed in their residence by trained staff using computer assisted interviews. In 2001 there were 33,326 sample adults who completed the survey (response rate 73.8%)^19^. All participants provided informed consent and the Research Ethics Review Board at the National Center for Health Statistics approved the study. All data collection and interaction with participants were conducted in accordance with relevant human subject guidelines mandated by the US Federal Government including confidentiality as specified by section 308(d) of the US Public Health Service Act (42 USC 242 M)(d). Research staff also sign a semiannual compliance certificate documenting compliance with nondisclosure policies.

No additional review was obtained for the present study because it involved publicly available data lacking identifying information.

### Social relationship assessments

The 2001 survey included several questions about social participation and one question on received social support. Received social support was assessed by the question “how often do you get the social and emotional support you need? Always, usually, sometimes, rarely, never.” Frequencies for the rarely/never categories were small and so were combined for analysis. We created a composite social integration variable based on yes/no responses to eight questions (range 0–8). Four questions assessed recent (past 2 weeks) contact with 1) friends or 2) relatives, either a) over the telephone or b) in person, excluding persons living with the respondent. Three other questions assessed whether participants attended a group social activity, a religious service, or went out to eat in the last two weeks. The final social integration item was marital status, defined as whether respondents were married/cohabiting or not. The two lowest social integration categories were too small to analyze separately (1.5–3% of respondents across racial and ethnic groups). To produce more stable estimates across racial and ethnic subgroups we collapsed social integration scores into 4 categories reflecting 0–3, 4–5, 6 and 7–8 social contacts. This provided 116, 437 and 84 mortality events in the 0–3 social integration category for Black, White and Hispanic participants, respectively. Similar categorizations are reported in the literature^20^ and below we describe sensitivity analyses using alternate social integration categories.

### Sociodemographic and health measures

Participants indicated whether they were of Hispanic ethnicity and whether they identified as Black or White race. Socioeconomic status measures included education (less than high school, high school, some college, college or higher), workforce status (employed, retired, out of work, never worked), wealth (home tenure; own versus rent/other) and annual family income (<$20,000; $20–34,999; $35–64,999; ≥$65,000). These represent a broad range of resources that capture human and material SES resources^18^. Other measures included age, sex, diagnosed chronic disease (cancer, heart attack, coronary heart disease, other heart disease, stroke; summed and scored none/one or more) and current smoking status (yes, no). Smoking status had 83 missing values that were singly imputed to nonsmoking status. Analyses with casewise deletion of these 83 participants were virtually identical to those reported below (data not shown).

### Mortality ascertainment

The National Center for Health Statistics linked baseline NHIS interviews to the National Death Index through December 31, 2011^17^. Of the 31,355 sample adults eligible for vital status ascertainment, 3,807 died over the 10-year follow up. We restricted our analyses to participants who described themselves as non-Hispanic Black, non-Hispanic White or Hispanic (*N* = 30,249). Missing data on social relationship assessments and other covariates reduced the sample size to 29,515 (3,557 deaths).

### Analytic approach

We used Cox proportional hazards models to estimate mortality risk as a function of social integration and social support. We estimated this association separately for Black, White and Hispanic participants. Since we did not assume a linear form in our social relationship predictors, we dummy coded both social relationship variables. All models incorporated the complex survey design including weights created for the ascertained vital status subgroup^21^. These weights adjust for non-response, oversampling of subgroups (e.g., persons 60 years of age and over) and permit nationally representative estimates^19^. Participants who were alive at follow up were censored. We used attained age as the time scale^22^ and stratified models by 5-year birth cohorts^23^,^24^. To fulfill proportional hazards assumptions we further stratified our survival models by sex, education, chronic disease and home ownership. Stratification accommodates covariates with non-proportional hazards by pooling hazards across levels of the stratification variables. This controls for but does not explicitly model the hazards associated with the stratification variables^25^,^26^. The remaining variables (workforce status, smoking) were used as covariates. Participants alive at the end of follow up were censored.

Regression models including household income persistently violated the proportional hazards assumption. Several specifications of income were explored without success so we excluded income from our main analytic flow and model diagnostics. However, we conducted several supplementary analyses to ensure our findings were robust to the exclusion of this important mortality determinant. We compared social relationship hazard estimates in the fully adjusted Cox model to hazards in models with multiply imputed income as a covariate^27^. We also included income as a covariate in a model with less restrictive assumptions, i.e., a logistic model (see below). These analyses showed that social relationship regression estimates were insensitive to adjustment for income.

### Sensitivity analyses

We examined whether poor health at baseline influenced both social relationships and subsequent mortality. We evaluated this possibility 1) by excluding participants who died within a year of the baseline interview and 2) by excluding participants with a diagnosed chronic disease at baseline. The proportional hazards assumption for the fully adjusted model was violated for both of these sensitivity analyses only among Hispanic participants. Visual inspection of the time by scaled Schoenfeld residual plots for the social relationship variables indicated a zero slope (and thus a consistent association over time) and therefore we did not pursue additional modeling solutions for these supplemental analyses among Hispanic participants^28^.

In another set of sensitivity analyses we compared mortality hazards using a 6-level social integration variable and we analyzed mortality without the time component, i.e., using logistic regression rather than time-to-event regression. For the logistic approach we used a generalized linear model with a Poisson family, a log link function and robust (linearized) standard errors^29^ to generate incidence rate ratios for this outcome. We used Stata MP 13.1 (Stata Corp, College Station, TX) and considered two-tailed *p*-values of 0.05 or less statistically significant.

## Results

Baseline characteristics of the sample are presented in Table 1. Of the 3,557 deaths over the ten year follow-up there were 540, 2,677 and 340 deaths for Black, White and Hispanic participants, respectively.

**Figure 1:**
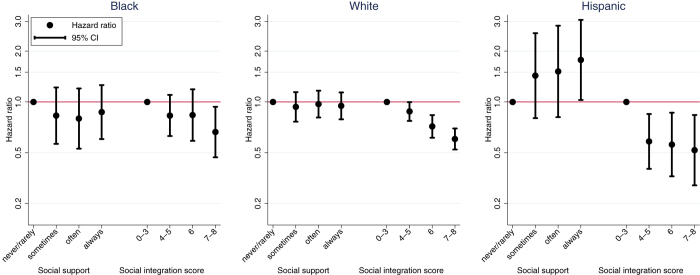
Hazard ratios for all-cause mortality by received social support and social integration for black, white and Hispanic participants, 2001 US National Health Interview Survey. Models are simultaneously adjusted for both social relationship variables as well as age, sex, education, home tenure (own vs. rent/other), workforce status (working [referent], retired, out of work, never worked), current smoking (yes, no) and diagnosed chronic disease (heart disease, stroke, and cancer).

In the fully adjusted model received social support was generally not associated with all-cause mortality for any of the three racial and ethnic groups (Fig. 1). In contrast, social integration was inversely associated with survival for all three groups, with the survival advantage restricted to the highest social integration group among Black participants (Fig. 1). Virtually identical hazard ratios were observed in models including multiply imputed income (Table 2) and similar patterns were observed when analyzing mortality in logistic models and when removing marital status from the social integration measure (data not shown).

### Sensitivity analyses

Analyses excluding those who died within a year of the interview and those who reported cancer, heart disease or stroke diagnoses at baseline revealed a generally similar direction and magnitude of hazard estimates for social support and social integration (Table 3). Thus, the association of social integration with ten year mortality did not appear to be explained by baseline health status or occult disease. Mortality hazards using a six category social integration variable were similar (Table 4).

### Evaluating the form of the social integration/mortality association

Given that social integration was associated with mortality within each racial/ethnic group we conducted exploratory analyses to examine the form of this association, i.e., whether it was threshold or linear. We compared the joint equivalence of the three highest social relationship categories using a Wald test. Among White participants the three highest social integration categories were statistically different from one another, *F*(2, 330) = 21.31, *p* < 0.001. This pattern, in combination with the consistently lower mortality risk for each of these categories relative to the referent, indicates a linear, dose-response association between social integration and mortality risk for Whites. For both Black and Hispanic participants we found no difference across the three highest social integration categories (for Blacks, *F*(2, 218) = 1.39, *p* = 0.252; for Hispanics, *F*(2, 208) = 0.18, *p* = 0.836, respectively). Although the equivalence of the three higher social integration groups was observed within both Black and Hispanic groups, the contrasts between these categories and the lowest social integration category suggest distinct forms of the social integration/mortality association. Among Blacks, these patterns indicate a flatter gradient whereby decreased risk is observed only for the highest social integration level (Fig. 1). The mortality hazard for Blacks at the highest social integration level (HR = 0.66, 95% CI = 0.47–0.94) was statistically equivalent to that of Whites at the same level (HR = 0.60, 95% CI = 0.52–0.70), Cochran’s Q = 0.25, *p* = 0.62^6^.

Among Hispanics, the form resembles a threshold, where reduced mortality risk is observed for all persons with social integration scores above three but with comparably reduced risk across the higher social integration categories (Fig. 1). Consistent with this threshold interpretation, mortality hazards for Hispanic (HR = 0.58, 95% CI = 0.40–0.85) and White (HR = 0.88, 95% CI = 0.77–1.00) participants at the threshold stratum (i.e., 4–5 social ties) were not estimating a common parameter^6^, Cochran’s Q = 4.11, *p* = 0.043.

## Discussion

We evaluated whether the putatively linear association between social relationships and all-cause mortality^2^ is consistent across Black, White and Hispanic adults in the US. Social integration was associated with lower ten year mortality risk for each of these social groups whereas received social support was not. These associations were insensitive to adjustment for a broad set of SES measures, initial health status and smoking. Despite this consistency, the form of the social integration/mortality association varied by race/ethnicity; it was dose response for Whites whereas for Blacks the survival advantage occurred only at the highest social integration levels. For Hispanics, the survival advantage appeared in all groups above the lowest social integration category and this advantage was comparable across moderate to high levels of social integration.

This is the first study of which we are aware to model the social relationship/mortality association separately for Blacks, Whites and Hispanics in a contemporaneous nationally representative cohort. Our findings suggest that aggregate analysis of racially and ethnically heterogeneous samples masks potentially important variation in the association of social integration with mortality^16^. Analogously, extreme group contrasts (i.e., contrasting the highest and lowest social integration categories) also obscures different forms of the mortality gradient for Black, White and Hispanic participants. The distinct patterns observed here qualify summary claims of linearity for the social integration/mortality association, claims which are partially based upon extreme group comparisons^2^. In addition, these data extend the literature by explicitly demonstrating that social integration, but not received social support, predicts mortality risk after adjustment for important covariates (SES, smoking)^30^.

Although these data are suggestive they cannot definitively establish a linear form for this association among Blacks. For example, although mortality hazards were flatter among Blacks detecting differences between hazard slopes for Whites and Blacks may only be possible with extremely large samples, particularly given the lack of statistical power for such comparisons^6^. In contrast, the threshold pattern among Hispanics was statistically distinct and to our knowledge this nonlinear association for social integration is novel in the literature. Because the survival advantage of social integration was realized for approximately 89% of Hispanics in our nationally representative sample we conjecture that it could indicate an “upstream” determinant of the Hispanic mortality advantage^31^. Conversely, the somewhat weaker association of social integration with mortality among Blacks may represent another form of health disparity.

Our work is contextualized by acknowledging several limitations. We modeled survival risk within each social group separately rather than use interaction terms for race/ethnicity in an aggregated sample. This is an indirect approach but avoids the implausible assumption that SES indicators are equivalent across race/ethnicity^32^,^33^. Our study measured social relationships at only one time and thus may misclassify social relationship resources over the course of follow up. However, social integration is fairly stable over time^34^ and the prognostic value of social support for mortality is not improved by repeated assessments^35^. Although we had a broad set of SES indicators we lacked area-level SES characteristics that are associated with mortality^36^,^37^,^38^. Conversely, available evidence suggests that area-level SES does not moderate the association of social relationships with mortality^39^ and that the addition of multiple social relationship assessments does not appreciably change their prognostic value^35^.

These issues notwithstanding, what might be driving these more nuanced patterns? These finding highlight the need for more basic research in this area as no theoretical framework in relationships and health that we know of makes predictions about distinct racial and ethnic groups. Conversely, extant theoretical models^1^,^4^ do make putatively universal claims for the salutary associations of social relationships – therefore, these data suggest caveats for such assertions. This study can rule out some simple statistical/methodological explanations as each group had similar distributions of social integration resources and mortality rates were comparable, albeit lower, for Hispanics. Presuming these patterns are replicated, future work might examine conceptual reasons for these racial and ethnic differences such as a reduced ability to effectively deploy social resources to health-related domains^40^ or in terms of differences in the importance assigned to social roles by race/ethnicity^41^.

Recent theoretical arguments suggest that received support may have more variable links to health because it is context specific^3^. That is, people can receive both effective and ineffective support from others which can obscure links to health unless such influences are modeled^42^. Consistent with this suggestion, other nationally representative data show that high levels of received support were related to greater mortality when it was perceived as low in responsiveness^43^. The finding that the highest level of received support was associated with greater mortality for Hispanics might mean that they are receiving less effective support. Future work examining ethnic differences in support responsiveness or negative social exchanges might help explain this pattern. Nevertheless, there might be a simpler statistical explanation. Because social integration and social support are positively correlated among Hispanics (*r* = 0.24) having both variables in the regression together might artificially reverse the social support coefficients^44^,^45^. In post hoc analyses we compared the hazards for emotional support with and without adjustment for social integration. The statistical association of emotional support with survival attenuated to nonsignificance when omitting social integration despite a more favorable estimation context, i.e., a model with fewer estimated parameters and more social relationship variance to explain. In addition, the emotional support hazard ratios were even weaker in models that excluded SES and/or the stratification variables (data not shown). Thus, one parsimonious explanation is that the apparent disadvantage of high support among Hispanics is a statistical artifact, although future work that directly models important social processes as a function of ethnicity would be needed.

In sum, this study replicates a widely reported pattern of higher social integration predicting decreased mortality risk. We also show for the first time that this association generalizes to a contemporaneous population-based cohort of Black, White and Hispanic participants followed for ten years. This association was robust to control of a variety of SES indicators, a pattern consistent with assertions that social relationships are an element of SES^18^,^46^,^47^ rather than a “downstream” consequence of SES^4^. The differing forms of the social integration/mortality association across racial and ethnic groups, should they prove robust in future research, reveal potential limitations of extant social relationship theory and indicate the need for additional research addressing the unique patterning and potency of social relationships in diverse samples.

## Additional Information

**How to cite this article**: Barger, S. D. and Uchino, B. N. Racial and Ethnic Variation in the Association of Social Integration with Mortality: Ten-year Prospective Population-based US Study. *Sci. Rep.* **7**, 43874; doi: 10.1038/srep43874 (2017).

**Publisher's note:** Springer Nature remains neutral with regard to jurisdictional claims in published maps and institutional affiliations.

## Figures and Tables

**Table 1:** Participant Characteristics by Race/Ethnicity, 2001 National Health Interview Survey.

Characteristic	Black (*n* = 4,201)		White (*n* = 20,217)		Hispanic (*n* = 5,097)		Total (*n* = 29,515)	
	No. of Participants	%	No. of Participants	%	No. of Participants	%	No. of Participants	%
Age, weighted mean, y (SD)	41.9	18.2	46.3	16.6	39.6	19.7	45.1	17.5
Female	2,645	56	11,273	52	2,847	51	16,765	52
Education level								
Less than high school	1,074	24	2,684	13	2,329	44	6,087	18
High school diploma	1,279	31	6,017	30	1,201	24	8,497	30
Some college	1,266	31	6,142	30	1,098	22	8,506	30
College graduate or higher	582	14	5,374	26	469	10	6,425	23
Workforce status								
Working	2,622	65	13,042	67	3,319	67	18,983	67
Retired	517	10	3,857	16	389	7	4,763	14
Out of work	837	20	2,777	14	827	16	4,441	15
Never worked	218	6	528	3	559	10	1,305	4
Unknown	7	0	13	0	3	0	23	0
Own home	1,853	51	14,453	77	2,194	50	18,500	71
Household income, dollars								
<20,000	1,332	24	3,393	12	1,620	23	6,345	15
20,000–34,999	789	18	3,198	14	1,046	20	5,033	15
35,000–64,999	758	20	4,710	24	1,009	23	6,477	24
≥65,000	447	15	4,744	28	479	14	5,670	25
missing	875	22	4,172	21	943	20	5,990	21
Chronic diseases^a^								
None	3,555	87	15,919	80	4,653	92	24,127	82
One	480	10	3,064	14	331	6	3,875	12
Two or more	166	3	1,234	6	113	2	1,513	5
Current smoker	985	22	5,010	24	919	17	6914	23
Social support								
Never/rarely	316	7	1,129	5	399	7	1,844	5
Sometimes	734	16	2,523	11	755	13	4,012	12
Usually	1,238	29	7,455	37	1,431	28	10,124	35
Always	1,913	48	9,110	47	2,512	51	13,535	48
Mean (95% CI)^b^	4.2	4.1, 4.2	4.2	4.2, 4.3	4.2	4.2, 4.2	4.2	4.2, 4.2
Social integration								
0–3 ties	503	11	1,640	7	631	11	2,774	8
4–5 ties	1,158	26	5,092	23	1,314	24	7,564	23
6 ties	911	21	5,195	25	1,080	21	7,186	24
7–8 ties	1,629	42	8,290	45	2,072	44	11,991	44
Mean (95% CI)^c^	5.8	5.8, 5.9	6.1	6.0, 6.1	5.9	5.8–5.9	6.0	6.0, 6.0
Deceased at follow up	540	10.5	2,677	11.1	340	5.9	3,557	10.5

^a^Reported diagnosis of cancer, heart attack, coronary heart disease, other heart disease, or stroke.

^b^Scored 1 = never, 5 = always.

^c^Scores range from 0–8 ties.

Percentages and means are weighted to estimate the civilian noninstitutionalized US population. SD, standard deviation; CI, confidence interval.

**Table 2:** All-Cause Mortality Hazard Ratios by Race/Ethnicity for Received Support and Social Integration Controlling for Multiply Imputed Family Income.

	Black (*n* = 4,201)			White (*n* = 20,217)			Hispanic (*n* = 5,097)		
	HR	95% CI	*P* Value	HR	95% CI	*P* Value	HR	95% CI	*P* Value
Social support									
Never/rarely	(referent)			—			—		
Sometimes	0.82	0.56, 1.20	0.314	0.94	0.77, 1.14	0.514	1.57	0.88, 2.78	0.124
Usually	0.79	0.53, 1.17	0.242	0.98	0.81, 1.17	0.797	1.61	0.86, 3.01	0.135
Always	0.88	0.61, 1.26	0.480	0.96	0.80, 1.15	0.641	1.92	1.11, 3.33	0.020
Social integration									
0–3 ties	(referent)			—			—		
4–5 ties	0.83	0.62, 1.10	0.192	0.88	0.78, 1.00	0.052	0.59	0.41, 0.85	0.005
6 ties	0.83	0.58, 1.18	0.303	0.73	0.62, 0.85	<0.001	0.58	0.38, 0.90	0.015
7–8 ties	0.67	0.48, 0.95	0.024	0.61	0.53, 0.71	<0.001	0.56	0.36, 0.89	0.013

Note: HR = hazard ratio; CI = confidence interval. Survival models are stratified by 5-year age cohort, sex, education, chronic disease, home tenure (own vs. rent/other) and covary workforce status (working [referent], retired, out of work, never worked), smoking (yes, no) and multiply imputed family income ($0–$19,999 [referent]; $20–$34,999; $35–$64,999; $65,000 or more per year). Analyses utilized the five imputed income files created by the data producer (National Center for Health Statistics^27^).

**Table 3:** All-Cause Mortality Hazard Ratios [95% CI] for Received Support and Social Integration by Race/Ethnicity Excluding Early Mortality and Participants with Chronic Disease at Baseline.

	Black (*n* = 4,201)			White (*n* = 20,217)			Hispanic (*n* = 5,097)		
	HR	95% CI	*P* Value	HR	95% CI	*P* Value	HR	95% CI	*P* Value
**Exclude early mortality**									
Social support									
Never/rarely	(referent)			—			—		
Sometimes	0.89	0.58, 1.37	0.598	0.88	0.72, 1.09	0.247	1.64	0.85, 3.13	0.137
Usually	0.84	0.55, 1.30	0.434	0.95	0.78, 1.15	0.579	1.65	0.83, 3.28	0.150
Always	0.92	0.61, 1.39	0.691	0.88	0.73, 1.07	0.201	2.02	1.08, 3.77	0.028
Social integration									
0–3 ties	(referent)			—			—		
4–5 ties	0.87	0.64, 1.18	0.357	0.91	0.79, 1.04	0.172	0.59	0.40, 0.89	0.012
6 ties	0.91	0.63, 1.31	0.610	0.76	0.64, 0.90	0.001	0.54	0.33, 0.86	0.011
7–8 ties	0.72	0.50, 1.04	0.082	0.63	0.54, 0.74	<0.001	0.51	0.31, 0.85	0.010
**Exclude any baseline chronic disease**									
Social support									
Never/rarely	(referent)			—			—		
Sometimes	1.17	0.63, 2.17	0.616	0.99	0.73, 1.35	0.970	1.52	0.73, 3.16	0.264
Usually	1.26	0.65, 2.44	0.489	1.04	0.78, 1.39	0.797	1.63	0.77, 3.43	0.197
Always	1.55	0.84, 2.86	0.157	1.01	0.76, 1.34	0.946	1.76	0.92, 3.37	0.087
Social integration									
0–3 ties	(referent)			—			—		
4–5 ties	0.79	0.54, 1.14	0.198	0.97	0.81, 1.18	0.782	0.67	0.43, 1.06	0.084
6 ties	0.61	0.39, 0.97	0.035	0.74	0.59, 0.95	0.017	0.57	0.34, 0.96	0.036
7–8 ties	0.51	0.35, 0.76	0.001	0.65	0.52, 0.80	<0.001	0.59	0.34, 1.02	0.058

Note: HR = hazard ratio; CI = confidence interval. Survival models are stratified by 5-year age cohort, sex, education, chronic disease, home tenure (own vs. rent/other) and covary workforce status (working, retired, out of work, never worked) and smoking (yes, no). Early mortality is defined as within a year of the baseline interview. Baseline chronic disease was the presence of any diagnosed heart disease, stroke or cancer.

**Table 4:** All-Cause Mortality Hazard Ratios by Race/Ethnicity for Received Support and a 6-Level Social Integration Variable; 2001 US National Health Interview Survey.

	Black (N = 4,201)			White (N = 20,217)			Hispanic (N = 5,097)		
	HR	95% CI	*P* Value	HR	95% CI	*P* Value	HR	95% CI	*P* Value
Social support									
Never/rarely	(referent)			—			—		
Sometimes	0.83	0.56, 1.21	0.328	0.94	0.77, 1.15	0.529	1.44	0.80, 2.61	0.222
Usually	0.80	0.53, 1.20	0.282	0.98	0.81, 1.17	0.798	1.52	0.81, 2.88	0.192
Always	0.87	0.60, 1.25	0.448	0.96	0.80, 1.15	0.652	1.78	1.02, 3.10	0.041
Social integration									
0–3 ties	(referent)			—			—		
4 ties	0.86	0.61, 1.22	0.407	0.94	0.80, 1.10	0.415	0.61	0.37, 1.00	0.048
5 ties	0.80	0.58, 1.11	0.180	0.84	0.73, 0.96	0.010	0.57	0.38, 0.84	0.005
6 ties	0.84	0.59, 1.19	0.317	0.71	0.61, 0.83	<0.001	0.56	0.36, 0.86	0.009
7 ties	0.72	0.50, 1.02	0.068	0.64	0.55, 0.74	<0.001	0.53	0.32, 0.88	0.014
8 ties	0.56	0.34, 0.92	0.021	0.54	0.45, 0.64	<0.001	0.49	0.28, 0.87	0.015

Note: HR = hazard ratio; CI = confidence interval. Survival models are stratified by 5-year age cohorts, sex, education, chronic disease, home tenure (own vs. rent/other) and covary workforce status (working, retired, out of work, never worked) and smoking (yes, no).
